# 早期非小细胞肺癌的微创介入治疗

**DOI:** 10.3779/j.issn.1009-3419.2020.101.01

**Published:** 2020-06-20

**Authors:** 天煜 何, 金林 曹, 金明 徐, 望 吕, 坚 胡

**Affiliations:** 310003 杭州，浙江大学医学院附属第一医院胸外科 Department of Thoracic Surgery, the First Affiliated Hospital, Zhejiang University, Hangzhou 310003, China

**Keywords:** 肺肿瘤, 微创, 介入治疗, Lung neoplasms, Minimally invasive treatment, Interventional treatment

## Abstract

肺癌是目前全球最常见的癌症和癌症死亡的主要原因，其中非小细胞肺癌（non-small-cell lung cancer, NSCLC）约占肺癌总数的85%。随着计算机断层扫描（computed tomography, CT）等影像学筛查手段得到不断普及，肺癌的病理类型从以往以晚期中央型肺鳞癌为主，转变为现在的以早期周围型磨玻璃样结节等为表现的肺腺癌为主。肺癌的早诊早治有着重要意义，而微创介入技术的不断发展完善，使得肺癌治疗有了更多的选择，例如立体定向放射、经皮穿刺消融、支气管介入等。本文将就目前临床常见的这些微创介入治疗的作用原理、优势、不足及展望做一评述。

## Abstract (en)

Lung cancer is the most common cancer and the leading cause of cancer death in the world, among which non-small cell lung cancer (NSCLC) accounts for about 85% of the total lung cancer. With the widespread of computed tomography (CT) and other imaging screening methods, the pathological types of lung cancer have changed from central squamous cell carcinoma to the early-stage lung adenocarcinoma, which is manifested as isolated pulmonary nodules and ground glass nodules on CT. Early diagnosis and treatment of lung cancer is of crucial clinical significance, and the continuous development and improvement of minimally invasive interventional techniques provide more options for lung cancer treatment, such as stereotactic radiation, percutaneous ablation, and bronchial intervention. This paper will make a review on the principle, advantages, disadvantages and prospects of minimally invasive interventional therapy commonly used in clinical practice.

肺癌是目前全球发病率和死亡率最高的肿瘤类型（分别占发病总数的11.6%和癌症死亡总数的18.4%）^[1]^其中非小细胞肺癌（non-small cell lung cancer, NSCLC）约占肺癌总数的85%。随着计算机断层扫描（computed tomography, CT）等影像学筛查手段的普及，肺癌的病理类型已经发生了显著变化。以往肺癌病理类型以中央型肺鳞癌为主，且确诊时多已为晚期而失去根治机会，预后较差；目前腺癌的发病率明显上升，已经超过鳞癌成为最常见的肺癌类型，其在CT影像上多表现为周围型的孤立性肺结节、磨玻璃样结节等，且大多为早期，因而也具有更大的治疗价值。

目前，手术切除肿瘤仍然是可切除的早期NSCLC的首选，且解剖性肺叶切除优于楔形切除，Ⅰ期肿瘤可首选电视辅助胸腔镜手术切除（video assisted thoracoscopic surgery, VATS）。对于不愿意承担手术风险，或者手术风险非常高的患者，根治性放疗[包括立体定向放射治疗（stereotactic body radiation therapy, SBRT/SABR）或低分次照射高剂量放疗]多为非手术治疗的首选^[2]^。另外，微创介入技术的不断发展完善，使得肺癌的治疗具有了更多的选择，例如经皮消融治疗、光动力治疗等方案也逐渐成为临床上的可选方案，其同时也具有手术治疗所不具备的优势。本文将分别就这些目前临床上常见的肺癌的微创介入治疗的作用原理、优势、不足及展望做一评述。

## 1 SBRT

从前临床上早期无法进行手术切除的NSCLC患者往往需要接受传统放疗，而其效果往往难以令人满意，约有60%-70%的患者原发肿瘤难以得到长期控制，超过1/2的患者最终死于肿瘤进展，2年生存率低于40%^[3, 4]^。近十余年来SBRT的出现使超过90%的无法手术的肿瘤和Ⅰ期可手术的NSCLC的整体生存率较传统放疗得到极大的提高^[6]^，目前美国国立综合癌症网络（National Comprehensive Cancer Network, NCCN）、欧洲临床肿瘤协会（European Society for Medical Oncology, ESMO）、欧洲放射肿瘤学学会（European Society of Radiotherapy and Oncology, ESTRO）等肺癌临床指南已经将SBRT列为早期不可手术NSCLC患者的一线治疗方案。

SBRT采用高度精确的立体定向技术实现对目标区域的定位，确定放射野的数量和角度，将根治性的放射剂量通过外照射的方式聚焦到肿瘤部位，达到杀灭肿瘤的目的，同时放射剂量在目标几毫米外急剧减少，使附近重要结构免受辐射损伤^[4, 6, 7]^。有研究^[8]^表明SBRT可以引起免疫激活细胞的增加和免疫抑制细胞的降低。另有作者认为放疗可以通过上调抗原表达、抗原处理、主要组织相容性复合体（major histocompatibility complex, MHC）分子和共刺激信号，增加肿瘤免疫原性，通过改变细胞因子平衡来克服免疫抑制肿瘤的微环境，以及招募抗原提呈和免疫效应细胞，从而产生对肿瘤的免疫学作用^[9]^，这也揭示了SBRT与免疫治疗联合作用的新的研究方向。

目前已有大量相关的临床研究显示SBRT具有显著高于传统放疗的局部控制率和长期生存率，原发肿瘤以及局部受累肺叶的控制率高达90.2%-92.6%，3年总生存率达55.8%-95.0%，3年无病生存率达48.3%-86.0%（表 1）。也有研究^[10]^认为其对于早期肺癌的总生存率高于楔形切除，而可与标准肺叶切除相媲美，但目前仍没有证据支持可手术且风险较低的早期肺癌患者应接受SBRT治疗。

**1:** 早期NSCLC患者接受SBRT治疗总结 Reports of NSCLC patients receiving SBRT treatment

Author	Published time	Research type	Number of cases	Mean follow-up time (mo)	Primary tumor and local control rate	3-year OS (%)	3-year PFS (%)
Baumann P^[3]^	2009	Polycentric, Prospective	57	36	92% (3-year)	60	52
Timmerman R^[4]^	2010	Polycentric, Prospective	55	34.4	90.6% (3-year)	55.8	48.3
Haasbeek CJ^[5]^	2011	Retrospective	37 (Lung mun), 26 (pericardium)	35	92.6% (Lung mun), 90.2% (pericardium)	64.3	/
Chang JY^[6]^	2015	Prospective	58	40.2	/	95	86
OS: overall survival; PFS: progression-free survival; SBRT: stereotactic body radiation therapy; NSCLC: non-small cell lung cancer.							

关于SBRT的风险，Woody等^[11]^的一项研究显示鳞状组织亚型、年轻、较少的共病、较高的体重指数、较高的正电子发射断层摄影标准化摄取值、中央型肿瘤和较低的辐射剂量与局部治疗失败风险增加有关，其中鳞状细胞亚型是最强的预测因子，鳞癌患者SBRT的失败率明显高于非特殊的腺癌或NSCLC患者，3年累积局部失败率分别为18.9%、8.7%和4.1%。Baine等^[12]^的一项多机构分析也证实鳞癌具有更高的局部失败率、局部和远处复发和较差的总生存率。

SBRT的常见不良反应包括中央气道损伤、食管不良反应、血管损伤、胸壁疼痛、放射性肺炎等^[13, 14]^。对于不同部位的肺癌病灶，SBRT的治疗毒性反应不同。Timmerman等^[7]^进行的一项前瞻性Ⅱ期临床试验统计了70例接受高剂量SBRT（1周-2周内共三个阶段总计60 Gy-66 Gy剂量照射）的早期NSCLC患者，共有14例患者发生3级-5级毒性反应。在发生毒性反应的患者中，周围型肺癌接受治疗的患者2年免受严重毒性影响的比例为83%，而中央型肺癌接受治疗的患者仅为54%。作者认为高剂量SBRT不应用于中央气道附近肿瘤患者，因其毒性反应过大。

此外，也有SBRT用于局部进展、多原发灶肺癌患者治疗的报道，但目前为止都是回顾性的研究，这可能成为一个较有前景的研究方向。Paul等^[15]^的一项回顾性研究认为虽然对于 < 2 cm的肿瘤行SBRT与手术治疗的3年生存率无明显差异（SBRT *vs*手术：82.6% *vs* 86.4%），但对于 < 5 cm的肿瘤，手术治疗能明显提高患者的3年生存率（SBRT *vs*手术：80.0% *vs* 90.3%）。由于采用SABR治疗早期可手术肺癌将是肺癌护理的一个模式转变，因此在广泛应用于实践之前，尤其是应用于局部进展期肺癌患者，有必要进行进一步深入的评估。

## 2 经皮穿刺消融治疗

对于不适合或不愿意接受手术的患者，SBRT已经成为目前可行的替代方案，且已被证明对于Ⅰ期周围型肺癌可达到90%左右的5年局部控制率，并被指南推荐。然而，这种技术可能受限于中央型肺癌或毗邻胸壁的肺癌，且会导致更高的并发症发生率，主要包括肺出血、肝纤维化（≥3级）及胸壁疼痛。经皮消融治疗是另一种具有良好前景的微创疗法。目前常用的消融方式包括热消融（thermal ablation, TA）和不依赖热杀伤效应的冷冻消融（cryoablation, CRA），前者又包括射频消融、微波消融、激光消融。Uhlig等^[16]^回顾性研究了1, 102例行TA治疗及27, 732例行SBRT治疗的Ⅰ期NSCLC患者，结果显示TA和SBRT治疗后的总生存期无显著差异（1年：85.4% *vs* 86.3%，2年：65.2% *vs* 64.5%，3年：47.8% *vs* 45.9%，5年：24.6% *vs* 26.1%），可见TA具有不亚于SBRT的治疗效果。另外，有报道表皮生长因子受体（epidermal growth factor receptor, *EGFR*）突变的患者在接受EGFR-酪氨酸激酶抑制剂（tyrosine kinase inhibitors, TKIs）过程中，联合射频或微波消融局部耐药病灶，可以提高肿瘤局部控制率^[17]^。

### 2.1 射频消融（radiofrequency ablation, RFA）

射频消融治疗肺癌最早在1995年由Goldberg等^[18]^报道于兔肺癌模型，并于2000年由Dupuy等^[19]^首次报道应用于患者。临床上RFA通常是在CT或超声定位下确定穿刺部位与角度，然后将射频发生器连接的活动电极（active electrode）穿刺到肿瘤部位，释放出的高频电流再回流到放置在患者体表的离散电极（dispersive electrode），产生的热量使肿瘤组织局部温度升高到60 ℃以上，导致其蛋白质变性和凝固坏死^[20]^。

RFA主要适用于肺内的小到中等大的周围型肺癌，消融后局部进展率与肿瘤大小高度相关，肿瘤直径每增加一厘米，进展速度就显著增加^[28]^。在此，不得不提的是RFA存在一种吸热效应（heat-sink effect），其产生是由于靠近热源的大血管（通常 > 3mm）在进行消融过程中的热对流，导致传递到病变的热量损失，从而会影响RFA的效果^[29, 30]^。

RFA治疗Ⅰ期NSCLC的效果已经在一系列的临床研究中得到验证（表 2），其中治疗后1年生存率高达89%-92%，2年生存率达73%-78%，3年生存率达51.2%-59%，5年生存率则下降到27.1%-40%。Lam等^[23]^的临床研究认为RFA治疗后的总生存率不亚于SBRT。Ni等^[17]^的一项多中心回顾性研究显示对于持续EGFR-TKI治疗的低进展性病变，采取局部消融治疗分离的耐药部位，再继续使用原EGFR-TKI在这些患者中显示出良好的疗效，可取得额外10个月的疾病控制。

**2:** Ⅰ期非小细胞肺癌消融治疗结局总结 Reports of stage Ⅰ NSCLC patients receiving ablation treatment

Author	Ablation tpye	Number of cases	Follow-up time (mo)	OS (%)			
				1-year	2-year	3-year	5-year
Ambrogi MC^[21]^	RFA	57	47	89	/	59	40
Lam A^[22]^	RFA	967	62.5	89.8	/	51.2	27.7
Lam A^[23]^	RFA	335	42.0	89.3	/	52.7	27.1
Lencioni R^[24]^	RFA	106	24	92	73	/	/
Yang X^[25]^	MWA	47	30	89	62	43	16
Wang Y^[26]^	MWA	131	/	95.6	95.3	/	/
Lu Q^[27]^	MWA	69	/	75	54.2	29.2	/
RFA: radiofrequency ablation; MVA: microwave ablation.							

对于RFA的治疗后并发症，Kashima等^[31]^对420例患者共1, 403个肿瘤病灶进行1, 000次RFA治疗，结果显示有4例死亡（0.4%），其主要并发症发生率为9.8%，常见主要并发症有无菌性胸膜炎（2.3%）、肺炎（1.8%）、肺脓肿（1.6%）、出血后需要输血（1.6%）、气胸后需要胸膜固定（1.6%），较少见的有支气管胸膜瘘、臂神经损伤、肿瘤播散和膈肌损伤等，可见RFA是一种相对安全的手术，但它有一定的致命风险。

关于治疗后的复发，Beland等^[32]^回顾了79例接受RFA的NSCLC患者，结果显示性别、肿瘤位置和放射治疗与复发风险无关，肿瘤大小增加和分期增加与复发风险相关。这表明最常见的复发模式是局部复发，更积极的射频消融和辅助放疗可能改善预后。另一项研究^[33]^认为血清TNF-α、CCL-2、CCL-4水平的升高可能与RFA治疗不完全以及治疗后复发相关，而TNF-α、CCL-2、CCL-4的升高与骨髓源性抑制细胞（myeloid-derived suppressor cells, MDSCs）产生的NO升高有关。

### 2.2 微波消融（microwave ablation, MWA）

微波引起的离子振荡所产生的热量可导致肿瘤组织的热凝固和坏死，其程度与微波本身的强度和频率呈正比^[34-36]^。由于电磁波的特性，与射频消融相比，MWA允许通过不同组织连续、均匀地传导热量，从而实现更大的消融范围，更短的手术时间，并且其吸热效应更小，不受起搏器的干扰，无需在患者皮肤表面放置接地垫片^[26, 29]^。

相较RFA，MWA治疗Ⅰ期NSCLC的临床研究数据更少，但从收集到的一些研究中可见MWA的治疗效果与RFA相仿（表3），其中治疗后1年生存率达75.0%-95.6%，2年生存率达54.2%-95.3%，3年生存率则下降到29.2%-43.0%，且与 > 3.5cm的肿瘤相比， < 3.5cm的肿瘤局部复发率更低，总体生存率更高^[25, 37]^。对于局部复发的肿瘤，重复使用MWA也可以取得较高的控制率，且总体生存率与无进展生存率与无局部复发的患者相似^[37]^。也有研究^[38]^认为微波消融的术中疼痛显著低于射频消融。Li等^[39]^的一项临床研究显示，*EGFR*突变的NSCLC患者在MWA治疗后继续使用EGFR-TKI，可以显著延长患者的无进展生存率。

关于MWA的并发症，Carrafiello等^[40]^统计了16例接受MWA治疗的患者，其中气胸是最常见的并发症（25%），其他并发症包括胸腔积液和皮下肺气肿，但通常不需治疗性引流。

### 2.3 激光消融（laser ablation, LA）

激光消融是通过将光纤经皮置入肿瘤病灶，通过其导入的激光对周围肿瘤组织进行扩散消融，使其产生凝固坏死。LA目前在临床上较多应用于甲状腺及肝脏小结节的治疗，而在肺癌中应用的报道仍较少。Weigel等^[41]^统计了42例肺癌患者共计64个病灶行LA后，51个病灶（79.7%）局部肿瘤得到控制。在 < 1.5 cm的病灶中有9%的病灶在治疗后出现进展，但在较大的病灶中有超过11%的病灶在治疗后出现进展。

### 2.4 氩氦刀冷冻消融（cryosurgical ablation, CSA）

氩氦刀冷冻消融系统基于Joules-Thompson效应原理，通过对肿瘤组织进行“极低温-快速复温”的循环使其细胞受损。氩气暴露状态使其温度低至-150℃，冰晶在细胞内外液中形成。同时，肿瘤供血动脉的收缩，以及肿瘤细胞水肿引起的低氧状态可进一步加重其损伤。解冻时，通过引入高压氦气使温度升高到0℃或40℃^[29, 42]^，也有研究认为0℃的解冻温度可能具有更多潜在的好处^[43]^。

Yamauchi等^[44]^统计了160例接受CRA治疗的Ⅰ期肺癌患者，结果显示2年和3年生存率分别为88%和88%，总生存期中位数为68个月，无病2年和3年生存率分别为78%和67%，平均无病生存期为46.6个月。

需要一提的是，CRA同样也受到吸热效应的影响，但由于胶原结构具有抗冻融特性，可使相邻重要结构的损伤最小化，CRA在治疗中央型肿瘤（气管、支气管树和纵隔附近）方面可能具有特别优势^[30]^。同时，CRA对于治疗贴近胸壁、纵隔、骨骼肌的肿瘤具有优势，因其可以减轻治疗带来的痛苦^[45]^。Ferrer-Mileo等^[46]^统计了22篇相关文献，496例NSCLC患者，共计580处病灶后显示，CRA治疗后24 h平均疼痛评分下降62.5%，3个月下降70%，6个月下降80.9%。24 h阿片类药物需求下降75%，3个月阿片类药物需求下降61.7%，说明CRA对于减轻肺癌治疗后疼痛具有明显优势。

另外，目前已有多项临床前研究^[47, 48]^表明，联合冷冻消融和免疫检查点抑制剂可导致肿瘤的协同免疫反应，但其安全性和可行性以及潜在的协同治疗效应的分子机制仍待进一步研究。

## 3 光动力治疗（photodynamic therapy, PDT）

PDT是一种系统性治疗，对某些早期肿瘤可具有根治性效果，对无法手术的肿瘤，PDT可以显著延长生存期并提高生活质量。其采用的光敏剂（photosensitizer, PS）具有在肿瘤细胞内选择性浓聚的特点，并且可在特定波长的光照射下在特定位点被激活，激活后的PS可与氧气发生光动力反应，产生活性单线态氧（^1^O_2_），从而对局部肿瘤细胞产生细胞毒性作用，同时也可损伤微血管并介导局部炎症反应，起到对肿瘤组织的直接和间接杀伤作用。其具有局部组织毒性较小，全身不良反应轻微的特点^[49-51]^。但PDT对于转移性肿瘤效果欠佳^[50]^，也有研究者通过PDT诱导全身免疫反应来克服这一缺陷，认为相对于传统的细胞毒性药物只能诱导细胞凋亡，PDT可同时诱导细胞的凋亡和坏死，诱导坏死为主的治疗模式或许更能激活免疫炎症反应^[51, 52]^。但目前临床上更常见的则是通过联合化疗或放疗与PDT来降低毒副作用并弥补PDT的局限性^[53-55]^。Weinberg等^[55]^统计了9例因支气管内癌患者接受联合PDT和高剂量率腔内照射治疗，结果显示先接受高剂量率腔内照射治疗再接受PDT的7例患者分别获得84 d、3个月、15个月、2年、2年、5年的局部控制期。对于光敏剂的选择，有吲哚菁绿（indocyanine green）、酞菁锌（zinc phthalocyanine）、焦脱镁叶绿酸-α甲酯（pyropheophorbide-α methyl ester）、CuS纳米制剂、线粒体靶向蛋白钌（mitochondria targeted protein-ruthenium）、混合配体铱iii（mixed-ligand iridium iii）配合物等作为光敏剂的报道^[52, 53, 56-60]^，均证明具有不同程度的疗效，但目前仍缺乏不同光敏剂治疗效果的对比研究。

## 4 经支气管介入治疗

经支气管介入治疗主要用于中晚期中央型肺癌以及肺转移癌（乳腺癌、结肠癌、肾癌等）造成的恶性气道梗阻（central airway obstruction, CAO）的治疗，具体方法包括机械清创、气道扩张、支气管镜下消融治疗、支气管内支架置入、局部化疗药注射、支气管腔内放疗等^[61-66]^。目前关于经支气管介入治疗早期肺癌的报道较少。

电磁导航支气管镜（electromagnetic navigation bronchoscopy, ENB）是一种相对新兴的支气管镜技术。ENB采用对CT气道图像的三维重建和传感器定位技术，将可控内镜探头引导向周围肺病变，到达目标位置后结合支气管内超声（endobronchial ultrasound, EBUS）等技术可对病灶进行采样活检^[67]^。ENB目前主要应用于提高恶性病灶的诊断率^[68-71]^。Harms等^[72]^报道了采用ENB到达病变靶点后，通过EBUS在肿瘤内放置6-F近距离治疗导管，行近距离放疗，治疗5 d后随访（12个月），腔内超声和CT显示部分缓解，组织学显示肿瘤完全缓解，说明ENB应用于不适合常规支气管镜治疗的患者的方案是可行的。

## 5 总结与展望

NSCLC治疗后的生存率虽然已经有了一定的改善，但其仍然是威胁人类生命的最重大疾病之一。NSCLC的微创介入治疗对于延长早期肺癌及无手术指征的肺癌生存期具有重要意义。每种治疗手段都有其独特的优势，同时也具有相应的局限性，联合多种治疗手段，取长补短，或许能发挥出最佳的治疗效果。例如SBRT、RFA、MWA、CRA等多种治疗方法都显示出了与当前研究热门的免疫治疗联合应用的可能性^[8, 17, 39, 47, 48]^，因此具有进一步研究的前景。目前，许多介入及微创治疗仍处于探索及发展阶段，尚无一个相对规范的治疗标准，因此在不同单位之间推广具有较大困难。同时，一些治疗方案的疗效也欠缺多中心大样本的统计分析，因此其应用价值尚待进一步考证。相信经过将来更多机构投入对介入及微创治疗的研究，NSCLC的治疗将逐步微创化、规范化，也将进一步减轻患者的痛苦，改善患者的预后。
